# Mild Physical Activity Does Not Improve Spatial Learning in a Virtual Environment

**DOI:** 10.3389/fnbeh.2020.584052

**Published:** 2020-11-17

**Authors:** Tavor Ben-Zeev, Inbal Weiss, Saar Ashri, Yuval Heled, Itay Ketko, Ran Yanovich, Eitan Okun

**Affiliations:** ^1^The Mina and Everard Goodman Faculty of Life Sciences, Bar-Ilan University, Ramat Gan, Israel; ^2^The Gonda Brain Research Center, Bar Ilan University, Ramat Gan, Israel; ^3^The Paul Feder Alzheimer's Disease Research Laboratory, Ramat Gan, Israel; ^4^The Institute of Military Physiology, Israeli Defense Forces Medical Corps, Ramat Gan, Israel; ^5^Heller Institute of Medical Research, Sheba Medical Center, Ramat Gan, Israel; ^6^Department of Military Medicine, Faculty of Medicine, Hebrew University, Jerusalem, Israel

**Keywords:** spatial learning, exercise, VO_2max_, respiratory exchange rate, virtual reality, physical activity

## Abstract

It is well-established that physical exercise in humans improves cognitive functions, such as executive functions, pattern separation, and working memory. It is yet unknown, however, whether spatial learning, long known to be affected by exercise in rodents, is also affected in humans. In order to address this question, we recruited 20 healthy young male adults (18–30 years old) divided into exercise and control groups (*n* = 10 in each group). The exercise group performed three sessions per week of mild-intensity aerobic exercise for 12 weeks, while the control group was instructed not to engage in any physical activity. Both groups performed maximal oxygen uptake (VO_2max_) tests to assess their cardiovascular fitness at baseline and every 4 weeks through the 12 weeks of the training program. The effects of mild aerobic exercise were tested on performance in two different virtual reality (VR)-based spatial learning tasks: (1) virtual Morris water maze (VMWM) and (2) virtual Radial arm water maze (VRAWM). Subjects were tested in both tasks at baseline prior to the training program and at the end of 12 weeks training program. While the mild-intensity aerobic exercise did not affect subjects' VO_2max_ parameters, mean time to anaerobic threshold increased for the exercise group compared with control. No effect was observed, however, on performance in the VMWM or VRAWM between the two groups. Based on these results, we suggest that mild-intensity aerobic exercise does not improve spatial learning and memory in young, healthy adults.

## Introduction

Aerobic exercise is well-known to improve long-term spatial learning and memory tasks in rodents (Lee et al., 2012). While physical exercise has been shown to improve executive functions in humans (Ploughman, 2008), it is yet unknown whether physical exercise indeed benefits human long-term spatial learning and memory.

Spatial learning is the process in which information about the environment is encoded to allow navigation through space and recall the location of motivationally relevant stimuli (Tan et al., 2017). This form of learning is critically dependent on the integrity of the hippocampus, which is commonly divided into several sub-regions: Cornu amonis (CA) 1–3, dentate gyrus (DG), and the hilus (Witter et al., 2000) and parahippocampal regions such as the medial entorhinal cortex (MEC) (Moser et al., 2008). The cognitive map theory (Eichenbaum, 2015) proposes that the hippocampus and other parahippocampal regions in rodents represent content and locations within the environment, providing the basis for spatial memory and navigation. With respect to humans, the theory also suggests lateralization of hippocampal function, with the right hippocampus encoding spatial relationships and the left hippocampus storing relationships between linguistic entities (Iglói et al., 2010). Moreover, one or both hippocampi incorporate temporal information derived from the frontal lobes, which serves to timestamp each visit to a location, thus providing the basis for a spatial short-term working memory system (which can hold information from seconds and up to several minutes) (Burgess et al., 2002).

Insights from human neuropsychology, neuroimaging, and electrophysiology strongly suggest an evolutionary continuity spanning mammalian species and implicating the hippocampal formation and its cortical inputs in allocentric spatial processing in rodents, primates, and humans alike (Banta Lavenex et al., 2014; Hartley et al., 2014).

A variety of methodologies have been utilized to assess spatial learning in humans. These methods significantly vary in their neural basis (Barak et al., 2015). Moreover, most methods are not fully compatible with spatial navigation as studied in rodents and described above. For example, computer-based methods such as the path-integration (Holzschneider et al., 2012) and taxi driver (Caplan et al., 2003) tasks do not involve head-movements, suggesting that computerized spatial tasks do not involve head-direction cells. Other spatial learning methods do not involve the hippocampus or the EC, as in the case of the dot fixation task (Nagamatsu et al., 2013) and the 3D rotation tasks (Holzschneider et al., 2012), suggesting the improper use of tools to assess spatial learning in humans. In contrast, VR environments have been used to assess spatial learning and memory in humans (Xu et al., 2012; Snider et al., 2013) due to their capability to activate the cell assemblies involved in spatial learning as suggested in rodents, thus enabling to mimic real-world navigation. It is important to distinguish between VR tasks, which run on a computer screen, from tasks that use VR goggles or a head-mounted display. The terminology of VR for tasks that run on a computer screen is not accurate, and these tasks should be considered under the “computer-based methods” category, including the virtual Morris water maze (VMWM) and the virtual radial arm maze (VRAM), which run on a computer screen and have been used widely in human studies (Levy et al., 2005; Herting and Nagel, 2012; Possin et al., 2016; Schoenfeld et al., 2017; Piber et al., 2018; Woost et al., 2018).

Cardiovascular fitness is the ability of body organs to consume, transport, and utilize oxygen. The maximal volume of oxygen the body can consume and use during exercise is termed maximal oxygen uptake (VO_2max_). There are several physiological adaptations from endurance training, such as the improvement of lactate clearance/tolerance, local vascularity, oxygen utilization, and stroke volume (Leveritt et al., 1999). Fit people have higher VO_2max_, which enables them to use oxygen more efficiently. In order to develop and maintain cardiovascular fitness in humans, aerobic exercise is typically performed at an intensity of 60–90% of maximal heart rate and duration of 20–60 min in each training over several months (Barak et al., 2015). VO_2max_ is essentially a measure of the body maximum oxygen utilization. It is the absolute peak of oxygen uptake, and therefore, aerobic performance. VO_2max_ is typically expressed in ml/kg/min, or oxygen used per unit of bodyweight per unit of time (and is, therefore, relative to body mass) (Howley et al., 1995).

At present, it is believed that the mammalian brain exhibits persistent plasticity throughout all stages of life. Neuronal plasticity allows the central nervous system (CNS) to learn new skills, consolidate and retrieve memories, reorganize neuronal networks in response to environmental stimuli, and recover after lesions. Physical exercise affects plasticity by several mechanisms; at the cellular level, exercise enhances hippocampal cell proliferation, anti-apoptotic pathways, and neurogenesis in a brain-derived neurotrophic factor (BDNF) dependent manner (Lee et al., 2012). Exercise-induced newly formed neurons are preferentially activated during learning tasks as well as contribute to the degradation of previously obtained memories (Cassilhas et al., 2016). The proliferation of brain endothelial cells and angiogenesis increases throughout the brain due to physical activity. Exercise-induced angiogenesis, the formation of new blood vessels, subsequently increases the availability of oxygen and glucose to existing neural circuitry (Lee et al., 2012; Barak et al., 2015; Cassilhas et al., 2016). Overall, findings in human studies are consistent with research in rodents, suggesting that physical activity may provide lasting benefits for brain structure and function (Voss et al., 2013).

There is scientific evidence that aerobic exercise improves hippocampal memory tasks, such as visuospatial memory for relationships between landmarks on maps (Herting and Nagel, 2012). Moreover, MRI studies show that prefrontal and temporal gray matter volume increases after physical exercise (Voss et al., 2013) and is also associated with angiogenesis (van Praag, 2009). With respect to humans, due to the large variety of tasks designed to tease out the possible effects of cardiovascular fitness on spatial learning, careful interpretation of these studies is required. The complicated studies conducted to date are far from providing a clear-cut answer to the question of whether cardiovascular fitness enhances spatial learning. The most recent studies, which used the reliable and realistic task of the VMWM in human subjects to assess the impact of cardiovascular fitness on long-term spatial memory, also conducted spatial learning and memory tests using a 3D computer screen (Woost et al., 2018). Thus, it is impossible to currently determine whether and to what extent cardiovascular fitness affects long-term spatial learning and memory abilities in humans.

In order to address this question, we developed spatial cognition tasks that were modified for virtual reality (VR) goggles. This enabled us to assess the long-term spatial learning and memory capacity of human participants by examining their ability to return to the exact location of a fixed target goal in a large environment with extra-maze cues.

## Methods

### Ethical Approval

The study was approved by the Bar-Ilan University's Ethics Committee in accordance with the Declaration of Helsinki, and by the institutional review boards (IRBs) of the Israel Defense Force's Medical Corps (1529-15) and the Sheba Medical Center (3321-16). Prior to testing, subjects were explained on the study's aims and required to sign informed consent forms, which were approved by all three IRBs.

### Virtual Reality Apparatus

The cognitive tasks were programmed using the “Vizard 5 Virtual Reality” software (WorldViz, Santa Barbara, CA, USA). In the VMWM and the VRAWM tasks, subjects wore the Oculus Rift DK2 virtual reality goggles (Oculus VR, LLC, Irvine CA, USA). These goggles were utilized as a display that enabled subjects to see the room in a first-person perspective, as well as a rotating tool of the view, due to its capability to translate head movements in real-time to shifts of the viewpoint. A controller (X-Box, Microsoft) was used for navigation in the environment as well as for rotating the view left and right, in addition to the goggles' rotation. Prior to starting each experiment, instructions were presented and explained to the subjects.

### Experiment 1: Calibrating the VR Tasks

#### Subjects

Healthy volunteers (*n* = 40) were recruited among Bar-Ilan University's students (see Table 1 for Subjects' characteristics). Subject inclusion criteria were: (1) no wearing eyeglasses, (2) no history or current use of psychiatric drugs, (3) not diagnosed as having ADHD.

**Table 1:** Subjects' details in the VMWM and VRAWM tasks according to the number of trials.

**Test name**	**Number of subjects**	**Gender (F; M)**	**Age (mean ± sd)**	**Years of education (mean ± sd)**
VMWM 3 trials/day	10	5;5	22.0 ± 2.0	13.3 ± 0.9
VMWM 4 trials/day	10	6;4	23.7 ± 2.0	13.8 ± 1.3
VRAWM 2 cues	10	5;5	24.4 ± 2.1	14.4 ± 1.6
VRAWM 4 cues	10	6;4	22.0 ± 2.051	13 ± 0.0

*Summary of subjects' gender, age, and years of education in each test, as well as the number of subjects per test*.

#### Experimental Design

Participants were divided into four groups of ten subjects to calibrate the VR tests. Two groups performed the VMWM test while two groups performed the VRAWM. To calibrate the VMWM task, participants were divided into two groups; one performed the test with three trials/day, while the second group performed the test with four trials/day. To calibrate the VRAWM task, participants were divided into two groups; one group perform the task with 2 extra-maze cues, and the second group performed the task with four extra-maze cues. The subjects were initially instructed about the experimental goal, following which they were tested over several days (separately indicated for each experiment).

#### Cognitive Assessment

The participants were tested for baseline cognitive performance, using the following tasks and criteria:

#### VMWM

Long-term spatial memory was tested using the VMWM task, which consisted of a black and white arena (width: 51 × length: 51 × height: 27 m), with 3 different extra-maze cues on different walls and a dark circular area in it (diameter = 25 m). While participants could observe their environment at 360° by turning their heads and bodies while using the virtual reality goggles, direction of movement was determined solely by a hand-held controller. Subjects performed the task for 7 days with either three or four trials/day and had to find the hidden platform within 25 s. Upon successful completion of the task, a text box appeared on the screen informing the subject about finishing a successful trial. In the case of an unsuccessful trial, the participant was transferred to the platform location while being informed about it. At the target zone location, subjects had 10 s to study the spatial environment and memorize the platform's location. At the end of the last trial, subjects were informed that the test was done. The maze arena and the platform location did not change between the different days. On the first day, subjects preformed a familiarization phase that included 3 trials to find a visible red rectangular target within the VMWM arena. This phase is important for habituation to the VR goggles as well as for navigation with the controller (X-Box, Microsoft). It also allowed the participants to understand better the task due to its similarity to the test stage.

#### VRAWM

The VRAWM consisted of a black and white room (51 × length: 51 × height: 27 m). Different extra-maze cues were placed on different walls with a colored circular area in it (diameter = 12.5 m). Each arm (length = 4 m, width = 1 m) was surrounded by walls (height = 4 m) made of glass, which enabled the subjects to see the external cues. The subject performed the task for 5 days when one group had two cues and the second group had four cues; both groups had 25 s and three trials to find the hidden target zone. Once the subject found the target zone within the 25 s-time frame, an appropriate text box appeared on the screen. Otherwise, the subject was transferred to the desired location while being informed about it. Within the target zone, subjects had 10 s to turn in place in order to study and remember the specific location. At the end of the last trial, subjects were informed that the test was done. As with the VMWM, the maze arena and the platform location in the specific arm did not change between the different days.

### Experiment 2: The Effect of Mild-Intensity Aerobic Exercise on Spatial Learning and Memory

#### Subjects

Healthy subjects aged 18–30 years were recruited among Bar-Ilan University's students. Subjects were randomly divided into control and exercise groups, after dropouts, we end with seven subjects in the control group and 11 subjects in the exercise group. Subjects' anthropometric characteristics, including age, weight (kg), height (m) and BMI (kg/m^2^) are described in detail in Table 2. Subjects inclusion criteria were: (1) A score of 35–45 in the VO_2max_ test (average cardiovascular fitness), (2) a 19–28 BMI (body mass index) (Prentice and Jebb, 2001; Eknoyan, 2008), (3) Not wearing eyeglasses, (4) No history or current use of psychiatric drugs, and (5) Not diagnosed as having ADHD. All subjects had to make a general electrocardiogram (ECG) test by their physician as well as presenting an approval from the physician prior to initiating the study.

**Table 2:** Subjects' anthropometric characteristics (Value ± SD).

**Parameter**	**Control**	**Exercise**	**Significance**
Age (years)	24.9 ± 3.8	25.5 ± 1.6	NS
Weight (kg)	78.3 ± 17.8	78.4 ± 14.9	NS
Height (m)	1.78 ± 0.07	1.76 ± 0.04	NS
BMI (kg/m^2^)	21.6 ± 10.9	25.4 ± 4.9	NS

*Summary of subjects' age, weight (kg), height (m), BMI (kg/m^2^) between exercise and control group*.

To determine the number of participants in this study, we assessed the effect size and power of a similar study that examined the effect of high intensity interval training (HIIT) on pattern separation (Heisz et al., 2017). In this study, the effect size was 0.3 (small to moderate) with a power of 0.8. According to this analysis the sample size of the entire current study should be 24 subjects (12 per intervention group). However, because of constraints in recruiting participants, we were only able to recruit 20 subjects. As participants were randomly pre-assigned to experimental groups, and due to the fact that participants dropout was unequal between groups, the sample size was different between the two experimental groups.

#### Experimental Design

The study lasted for 14 weeks. In the first week, the subjects performed baseline assessment tests (physical and cognitive). Following the baseline assessment, the exercise group performed the training program (Table 3), with the control group instructed not to participate in any physical activity. Every 4 weeks, subjects in both groups performed the aerobic fitness physical assessment (VO_2max_ test). After the last VO_2max_ test on week 13 of the study, the subjects performed the final cognitive assessment tests on week 14 of the study.

**Table 3:** Exercise protocol.

**Week**	**Training session**
1–2	30 min walk
3–4	30 min of: (5 min walk- 5 min run) X3
5–6	30 min of: 10 min run- 10 min walk- 10 min run
7–8	30 min of: 10 min walk- 20 min run
9–10	30 min run
11–12	40 min run

*Twelve weeks training program, subjects performed 3 training sessions each week. Every 2 weeks there was an increment in the running time until the last week of the training program*.

#### Cognitive Assessment

Long-term spatial memory using (1) the VMWM task described in experiment 1, with three trials/day, and (2) The VRAWM. The VRAWM task described in experiment 1, with two trials/day and two different extra-maze cues. In both VMWM and VRAWM tasks, target location was not change between test days and between the baseline and post-intervention tests, in order to enable assessment of long-term spatial learning and memory. Utilizing the VR system enabled us to better mimic real-world navigation in a manner that spatial-learning related hippocampal and para-hippocampal cells, such as place cells, grid cells, and head direction cells, are involved (Moser et al., 2008).

#### Training Program

The training program consisted of 12 weeks that included moderate aerobic exercise sessions three times per week, starting at 2 weeks of 30 min walking sessions followed by 2 weeks training sessions of six intervals- 5 min walk and 5 min run for 30 min. Subjects instructed to keep a steady pace during all training sessions. The duration of the running interval increased every 2 weeks until reaching a continuous 40 min run (Table 3).

#### Cardiovascular Fitness Assessment

Treadmill incremental $\dot{\text{V}}$O_2max_ test analysis has been widely used to determine aerobic fitness (Fairshter et al., 1983; Weltman et al., 1990; Lourenço et al., 2011). Following 3 min of warm-up at 9–9.5 km·h^−1^, participants started the protocol at 10 km·h^−1^ with a fixed treadmill grade of 2%. This initial running speed was determined as the running speed reached in the previous familiarization sessions. After each 25-s interval, the speed was increased by 0.3 km·h^−1^ until volunteers reached exhaustion. Participants were encouraged to continue for as long as possible. After exhaustion, the participants underwent a 5-min recovery protocol during which the speed was decreased each minute from 100 to 60, 55, 50, 45, and 40% of the maximal achieved speed.

#### Physiological Measurements

The VO_2_, carbon dioxide output ($\dot{\text{V}}$co_2_), and respiratory exchange rate (RER) were measured breath-to-breath using a gas analyzer (CPX/D Med Graphics, St. Paul, MN, USA). The average values of each variable at every 25-s stage were used to analyze data and relate them to phase II of the $\dot{\text{V}}$O_2_ kinetics. Before each test, the analyzer was calibrated with a known gas mixture (12% O_2_ and 5% CO_2_), and the volume sensor was calibrated with a 3-L syringe. Heart rate (HR) was measured continuously via a Polar® heart monitor interface (Polar Electro Oy, Helsinki, Finland). The last completed stage was used to determine $\dot{\text{V}}$O_2max_, maximal achieved speed (s$\dot{\text{V}}$O_2max_), maximal $\dot{\text{V}}$co_2_($\dot{\text{V}}$co_2max_), maximal respiratory exchange ratio (RER_max_), and maximal HR (HRmax). The $\dot{\text{V}}$O_2max_ achieved was assessed in the presence or absence of the $\dot{\text{V}}$O_2_ “plateau” during the protocols.

#### Statistical Analysis

Performance in VMWM and VRAWM, $\dot{\text{V}}$O_2max_ and time to anaerobic threshold were all tested for normality using Shapiro–Wilk normality test prior to further statistical analysis. Analysis of the effect of mild intensity aerobic exercise on long term spatial learning and memory test was conducted using repeated measures (RM) Two-way ANOVA for the measured parameters (latency, success, path efficiency, speed and distance) for each group (control and exercise), and test length (7 days in the VMWM and 5 days in VRAWM(in both the baseline and post-intervention tests and the differences in latency between the baseline and post-intervention tests for both groups. Repeated measures (RM) Two-way ANOVA was also conducted to analyze improvements in fitness via $\dot{\text{V}}$O_2max_ and time to anaerobic threshold test for each group (control and exercise), between the 4 tests in the baseline test and after the training program test. Parametric *t*-test was used to analyze the differences between T1 and T4 in $\dot{\text{V}}$O_2max_ and time to anaerobic threshold test. For latency, success, path efficiency, speed and distance in the VMWM and VRAWM Sidak's multiple comparisons test was used. Sidak's multiple comparisons was also used in analyzing $\dot{\text{V}}$O_2max_ and time to anaerobic threshold. For parameters that did not show normal distribution we performed non-parametric Mann-Whitney test to analyze the difference between the final and first days of the tests to see improvement in learning and memory. Data is shown as mean ± SEM. All statistical analyses were carried out using GraphPad Prism Software.

## Results

**Figure 1:**
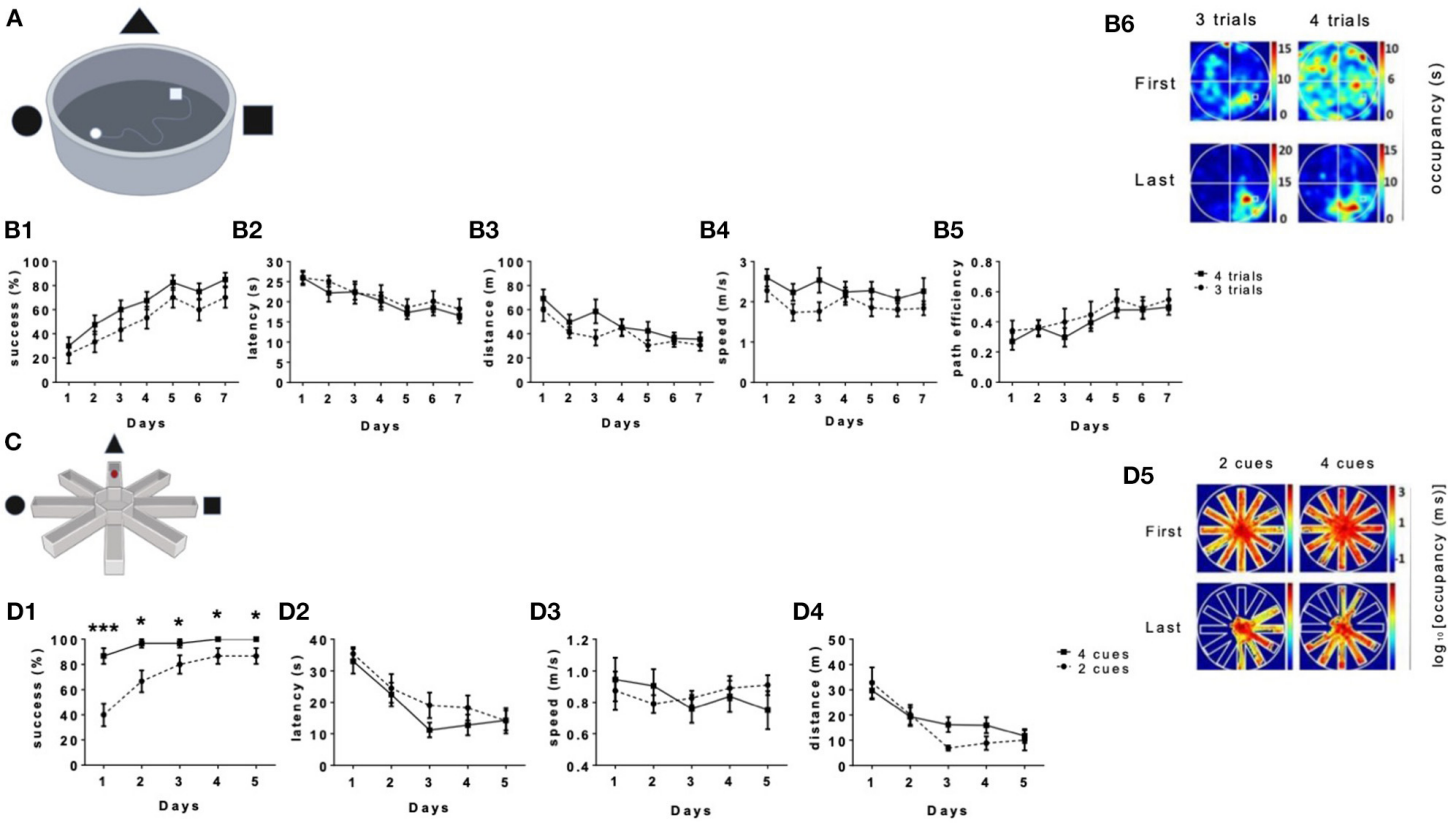
Long-term spatial learning in the VMWM according to the number of trials the number of cues. In the VMWM, both tests had 30 s per trial, which differed in the number of trials per day. **(A)** The platform's size and location are marked by a white square. Performance of the participants in this test was measured by: **(B1)** Success rates to find the target, **(B2)** Latency to reach the target, **(B3)** Total distance to reach the target, **(B4)** Movement speed and **(B5)** path efficiency. **(B6)** Occupancy plots show the first and last day of each test. The upper panel represents the first day and the lower panel represents the last day. Heat maps were calculated by the amount of time spent in seconds in the arena for the same day, normalized by the total time spent in the same position through all days of the task. Scale was determined by the maximal and minimal values. **(C)** The VRAWM tests had 30 s per trial which differed in the number of cues per day. Performance of the participants in this test was measured by: **(D1)** Success rates to find the target **(D2)** Latency to reach the target **(D3)**, movement speed **(D4)** Total distance to reach the target, and **(D5)** The occupancy plots are showing the first and last day of each test. Heat maps were calculated by the amount of time spent in seconds in the arena for the same day, normalized by the total time spent in the same position through all days of the task. Scale was determined by the maximal and minimal values. (*P < 0.05, ***P < 0.001).

The aim of this study was to address whether aerobic physical activity improves long-term spatial learning and memory in humans. While extensive data connects between physical activity and spatial learning and memory in rodents, the link in humans has not yet been causally established. To establish such a link in humans, we first generated and calibrated the VMWM and the VRAWM Since the MWM and RAWM spatial learning tasks were originally designed for rodents, we first conducted calibration tests to fit human subjects. It is common in most of the studies on rodents to use up to 4 trials per day to find the hidden target in the MWM (Figure 1A). we thus examined the performance of the participants when given three vs. four trials/day to assess the optimal protocol for human spatial navigation. Performance with both three and four trials/day resulted in similar learning curves with no significant differences in success in finding the platform (Figure 1B1). Both groups showed a main effect of days on latency to reach the platform [*F*_(1,18)_ = 14.08, *P* < 0.0001, Figure 1B2], total distance traveled [*F*_(1,18)_ = 10.90 *P* < 0.0001, Figure 1B3], speed of movement [*F*_(1,18)_ = 2.341, *P* = 0.0374, Figure 1B4], and path efficiency [*F*_(1,18)_ = 8.624, *P* < 0.0001, Figure 1B5]. Moreover, occupancy plots on the last day showed a higher presence in the target quadrant in both tests (Figure 1B6).

To calibrate the VRAWM (Figure 1C), 21 participants were recruited, and 20 of which have completed the tasks (Table 1). In the VRAWM, we examined the effect of using two vs. four extra maze cues on performance in this task. Subjects tested with four distal cues had a higher success rate to reach the platform compared with subjects tested with two distal cues, throughout the test (*P* = 0.0384, for the last day, Figure 1D1). In addition, there was a main effect of days on the latency to reach the platform (Figure 1D2) and total distance traveled (*P* < 0.0001, Figure 1D4). There were no significant differences in subjects' speed of movement between days (Figure 1D3). Occupancy plots of the first and last days (presented in logarithmic scale) show a reduction in the area covered between the first and last days. Moreover, the number of arms visited by the subjects was lower in the two distal cue test, thus more accurate (Figure 1D5).

### Time to Anaerobic Threshold but Not VO_2max_ Was Improved Following Mild Aerobic Exercise

Following calibrating the VR tasks (experiment 1), we have recruited an additional cohort of participants for the exercise group (*n* = 11) and the control group (*n* = 7) (experiment 2). There were no statistically significant differences in anthropometric characteristics [age, weight (kg), height (m) and BMI (kg/m^2^)] (Table 2).

**Figure 2:**
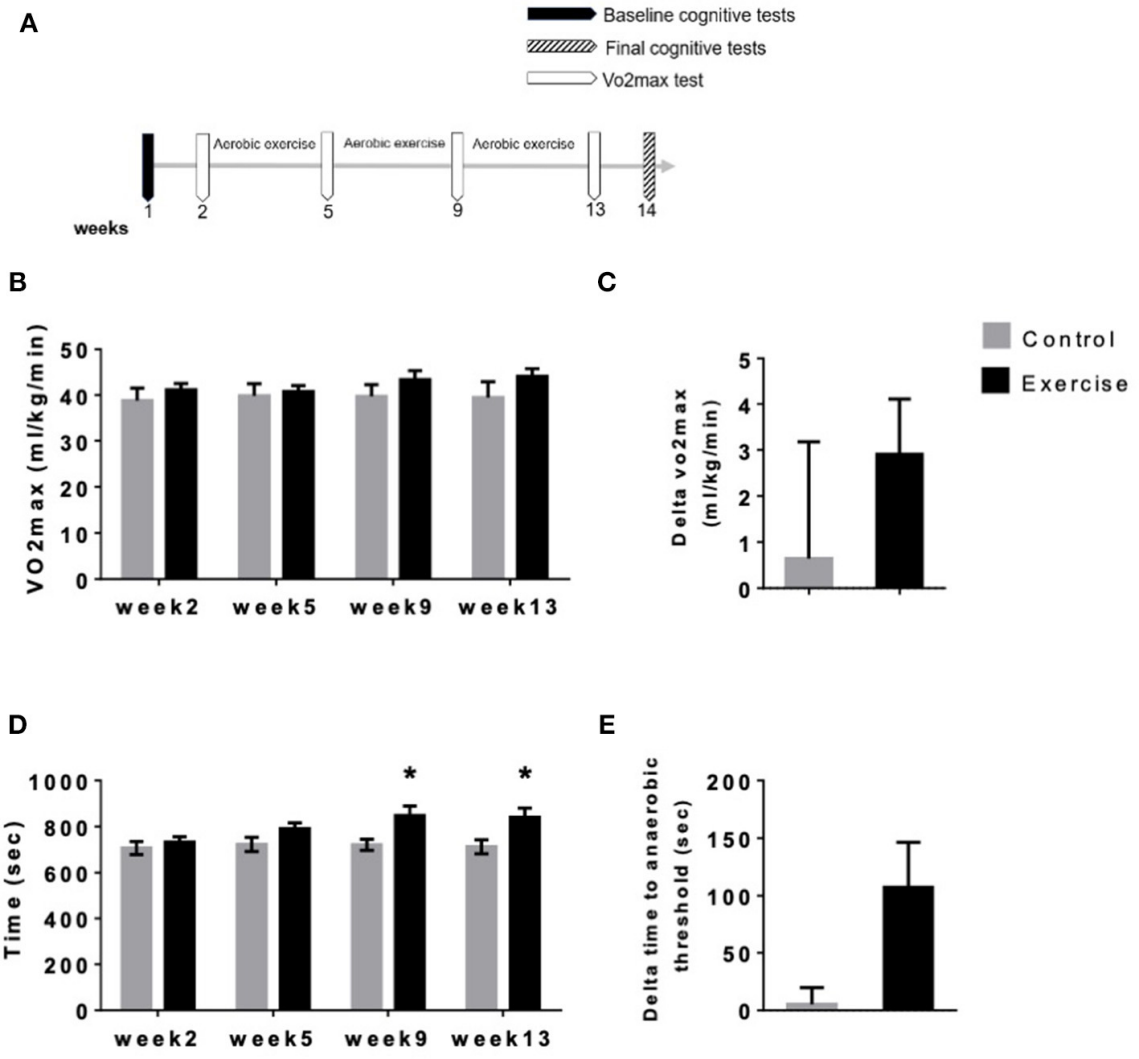
Time to anaerobic threshold but not VO_2max_ was improved following mild aerobic exercise. **(A)** Experimental scheme. **(B)** Vo_2max_ (mL kg^−1^ min^−1^) tests at 4 different time points spaced 4 weeks apart (T1–T4) **(C)** Vo_2max_ average improvements between T1 and T4 (*P < 0.05), **(D)** mean time to anaerobic threshold (sec) at four-time points spaced 4 weeks apart (T1–T4) and **(E)** time to anaerobic threshold improvements between T1 and T4 (*P < 0.05).

Eleven participants completed the 12 weeks of the aerobic exercise training program (Table 3, Figure 2A), while the seven control participants did not perform any physical activity exercises (Figure 2A). Both groups performed VO_2max_ (mL kg^−1^ min^−1^) tests at four different time points spaced 4 weeks apart (T1–T4, Figure 2B). When comparing the VO_2max_ of the exercise and control groups throughout the experiment, we observed no significant differences at T1 [41.16 mL kg^−1^ min^−1^ ± 1.36 and 38.9 mL kg^−1^ min^−1^ ± 2.72, respectively, *p* = 0.9360), T2, (40.75 mL kg^−1^ min^−1^ ± 1.3 and 39.84 mL kg^−1^ min^−1^ ± 2.64, respectively, *p* = 0.3673), T3 (43.36 mL kg^−1^ min^−1^ ± 1.96 and 39.72 mL kg^−1^ min^−1^ ± 2.56, respectively, *p* = 0.0726), or T4 (44.06 mL kg^−1^ min^−1^ ± 1.71 and 39.47 mL kg^−1^ min^−1^ ± 3.43, respectively, *p* = 0.0864) *F*_(1,16)_ = 1.059, *P* = 0.3188, Figure 2B].

Furthermore, no difference was observed between T4 and T1 between the two groups (2.905 mL kg^−1^ min^−1^ ± 1.209 and 0.6443 mL kg^−1^ min^−1^ ± 2.534, respectively, *p* = 0.3808, Figure 2C). Anaerobic threshold is defined by the increase of intensity during exercise and the rise in blood lactate concentration. Increment in blood lactate is an indication of an increase in glycogen metabolism. This increase in blood lactate is interpreted as a reflection of the onset of hypoxia in skeletal muscles, and the exercise intensity at which anaerobic metabolism complements the regeneration of ATP by aerobic metabolism is called the Anaerobic Threshold. Respiratory exchange ratio (RER) is a measure of anaerobic threshold, indicating the relationship between expired CO_2_ and inspired O_2_. RER is commonly used to indirectly determine the relative contribution of carbohydrate and lipids to overall energy expenditure. A high RER indicates that carbohydrates are being predominantly used, whereas a low RER suggests lipid oxidation, meaning that a high RER represents a more anaerobic energy production and a low RER represents a more aerobic energy production.

When comparing the time to anaerobic threshold between exercise and control groups, we see no statistically significant differences at T1 (733 s ± 23.32 and 707 s ± 28.27, respectively, *p* = 0.9360, Figure 2D) or T2 (791.5 s ± 24.9 and 722.9 s ± 30.98, respectively, *p* = 0.3673, Figure 2D). At T3 and T4, however, time to anaerobic threshold was higher in the aerobic exercise group compared with controls (T3; 848.2 s ± 41.53 and 721.6 s ± 24.13, *p* = 0.0417; T4; 840.4 s ± 40.17 and 712.7 s ± 30.58, *p* = 0.0394) *F*_(1,16)_ = 4.816, *P* = 0.0433, Figure 2D). Despite that a trend toward significance was observed between the two intervention groups (107.3 s ± 39.14 and 5.186 ± 14.60, respectively, *p* = 0.0621), this did not reach statistical significance (Figure 2E).

### Long-Term Spatial Learning and Memory Was Not Affected by Mild Aerobic Exercise

**Figure 3:**
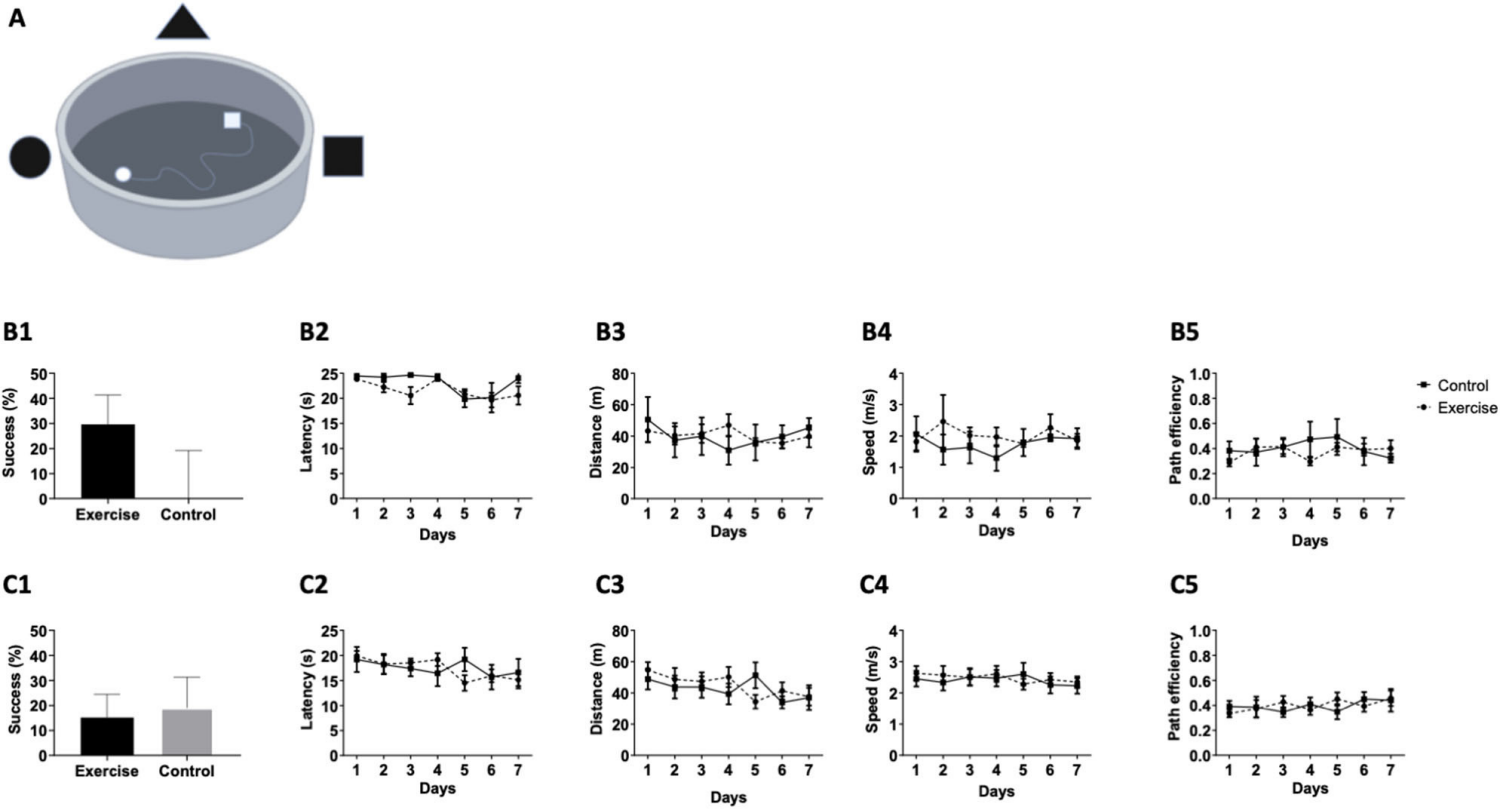
Mild aerobic exercise does not affect performance in the VMWM. Participants (n = 20) were randomized into exercise or control group (n = 10/group). The exercise group followed a training program that consisted of 3 running sessions/week, while the control group was guided not to exercise. (**A**) Prior to conducting the training program, the participants performed a virtual reality test in the VMWM apparatus to examine the baseline spatial learning abilities. The following parameters were measured: **(B1)** Success rates to find the target between day 1 and day 7, **(B2)** Latency to reach the target, **(B3)** Total distance to reach the target, **(B4)** Movement speed, and **(B5)** Path efficiency. At the end of the training program, participants were tested again in the same spatial learning VMWM task and were assessed for: **(C1)** Success rates to find the target between day 1 and day 7, **(C2)** Latency to reach the target, **(C3)** Total distance to reach the target, **(C4)** Movement speed, and **(C5)** Path efficiency.

At baseline, no statistical difference was observed between the exercise and control groups, as evident by the success rates in the VMWM (Figure 3A) between days 1 and 7 (*p* = 0.2364, Figure 3B1), latency [*F*_(1,16)_ = 1.331, *P* = 0.2754, Figure 3B2], distance [*F*_(1,16)_ = 0.005190, *P* = 0.9440, Figure 3B3], speed [*F*_(1,6)_ = 0.3841, *P* = 0.5493, Figure 3B4], and path efficiency [*F*_(1,16)_ = 0.2739, *P* = 0.6121, Figure 3B5). The last VMWM test was post-intervention of the aerobic exercise training program. No statistically significant differences in mean success between the control and exercise groups were observed. This was evident in success rate between day 1 to day 7 (*p* = 0.6679, Figure 3C1), latency [*F*_(1,16)_ = 0.01135, *P* = 0.9165, Figure 3C2], distance [*F*_(1,16)_ = 0.1355, *P* = 0.7176, Figure 3C3], speed [*F*_(1,16)_ = 0.0526, *P* = 0.8215, Figure 3C4], and path efficiency [*F*_(1,16)_ = 0.007322, *P* = 0.9329, Figure 3C5], suggesting that mild-intensity aerobic exercise does not affect long-term spatial learning and memory in the VMWM test.

**Figure 4:**
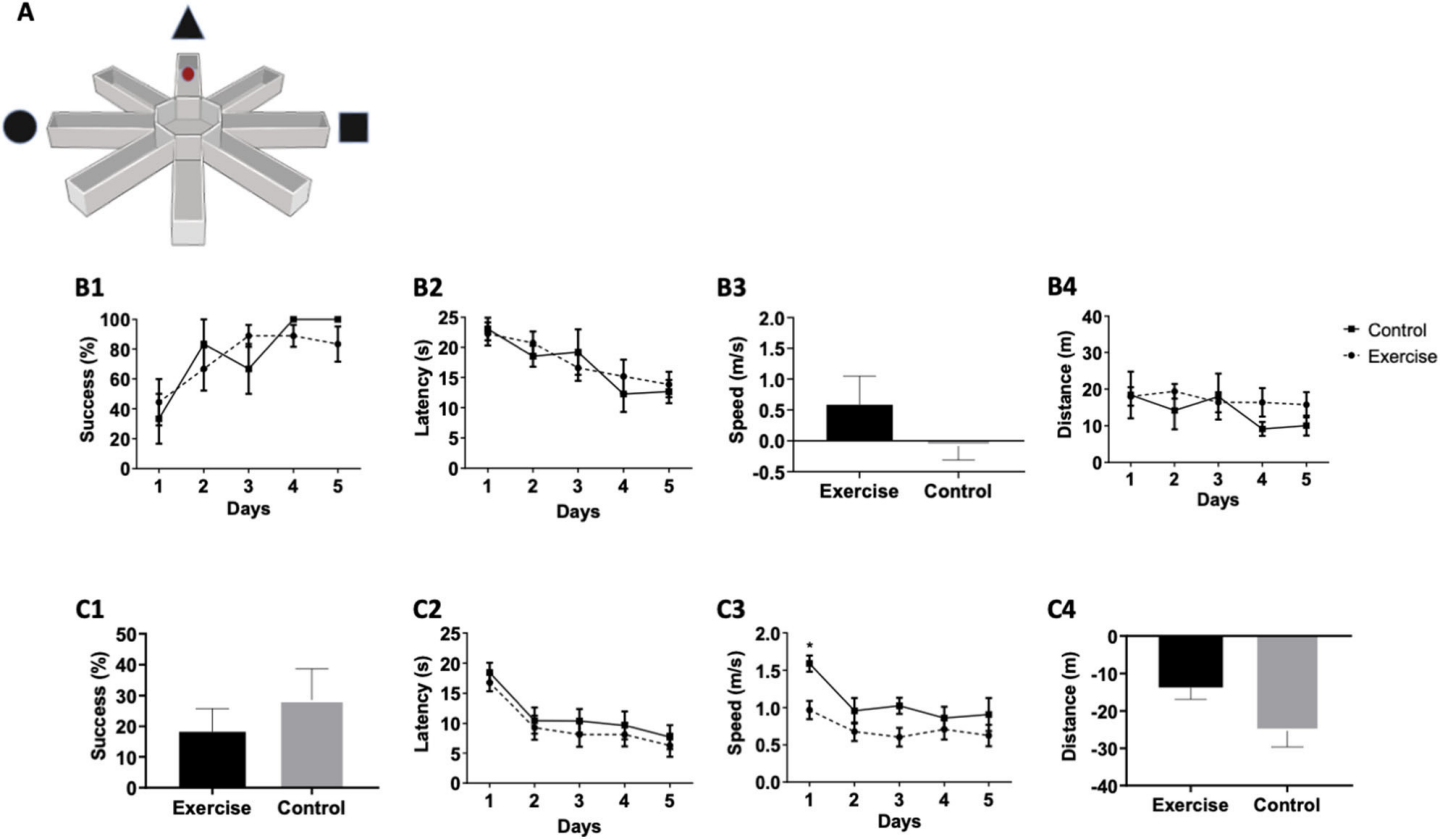
Mild aerobic exercise does not affect performance in the VRAWM. (**A**) In parallel to VMWM tests, the participants were also tested in the VRAWM aparatus. Participants performed a virtual reality test to examine the baseline spatial learning abilities. The following parameters were measured: **(B1)** Success rates to find the target, **(B2)** Latency to reach the target, **(B3)** Movement speed between day 1 and day 5, and **(B4)** Total distance to reach the target. At the end of the training program, participants were tested again in the same spatial learning VRAWM task and were tested for: **(C1)** Success rates to find the target between day 1 and day 5, **(C2)** Latency to reach the target, **(C3)** Movement speed, and **(C4)** Total distance to reach the target between day 1 and day 5. *P = 0.0172.

The VRAWM was conducted with two trials/day for 5 days (Figure 4A), with each lasting 25 s. Similar to the VMWM task, no significant differences were observed between the two groups in mean success [*F*_(1,16)_ = 0.02273, *P* = 0.8832, Figure 4B1], latency [*F*_(1,16)_ = 0.03205, *P* = 0.8615, Figure 4B2], distance [*F*_(1,16)_ = 0.4979, *P* = 0.4965, Figure 4B3], and speed between day 1 to day 5 (*p* = 0.3727, Figure 4B4). These results indicate that long-term spatial learning was similar between the two groups at baseline. Following 3 months of exercise, the final VRAWM test was conducted. No statistically significant differences were found between control and exercise groups, as evident in success rate between day 1 to day 5 (*p* = 0.6305, Figure 4C1), latency [*F*_(1,16)_ = 0.5042, *P* = 0.4879, Figure 4C2] and distance between day 1 to day 5 (*p* = 0.0556, Figure 4C4). While speed of movement in the task showed a significant effect [*F*_(1,16)_ = 5.181, *P* = 0.0369], it was mainly due to a significant difference on day 1 between the two intervention groups (*p* = 0.0172, Figure 4C3). Also, although the trend toward significance in the distance parameter between days 1 and 5 suggests that the exercise group traveled shorter distance to the target, compared with the control group, this did not corroborate with the overall success rates in this task. Overall, as success rates did not differ between the two intervention groups, suggesting that a mild-intensity aerobic exercise does not affect long-term spatial learning and memory in the VRAWM test.

As a result of the lack of significant differences, we have analyzed the differences within each experimental group between baseline and post-intervention tests.

In the VMWM task, both intervention groups showed no significant interaction between the intervention and days [Control: *F*_(6,48)_ = 0.6194, *P* = 0.7137; Exercise: *F*_(6,108)_ = 0.6686, *P* = 0.6752; Supplementary Figures 1A,B]. While in the control group there was a main intervention effect [*F*_(1,12)_ = 5.638, *P* = 0.0449], there was no main days effect [*F*_(3.333,26.67)_ = 0.6630, *P* = 0.5972, Supplementary Figure 1A]. In the exercise intervention group, two main effects were observed. A main intervention effect was found [*F*_(1,20)_ = 13.97, *P* = 0.0015], as well as a main days effect [*F*_(4.358,78.44)_ = 4.500, *P* = 0.0019, Supplementary Figure 1B). In the VRAWM, both intervention groups showed no significant interaction between the intervention and days [Control: *F*_(4,32)_ = 0.9371, *P* = 0.4551; Exercise: *F*_(4,72)_ = 1.269, *P* = 0.2901, Supplementary Figures 1C,D]. In the control group, while no intervention effect was observed [*F*_(1,12)_ = 4.720, *P* = 0.0616], a main days effect was observed [*F*_(2.588,20.71_ = 9.872, *P* = 0.0005, Supplementary Figure 1C]. In the exercise group, we observed both a main intervention effect [*F*_(1,20)_ = 12.35, *P* = 0.0025] and a main days effect [*F*_(2.725,49.05)_ = 14.05, *P* < 0.0001, Supplementary Figure 1D].

We also preformed correlation tests between VO_2max_, time to anaerobic threshold and latency in the last day post-intervention in both VMWM and VRAWM. No correlation was found between these fitness parameters and latency (Supplementary Figure 2).

## Discussion

The beneficial effect of physical exercise on several types of cognitive abilities is widely discussed in the scientific literature (Ploughman, 2008; Barak et al., 2015; Loprinzi, 2018). Few studies have investigated the effect of exercise on spatial learning in humans. In the current study, we used a VR maze task to measure and assess spatial learning and memory in a paradigm that is similar to real-world navigation in humans. Commonly used computer-based spatial learning tasks do not involve head-movements, suggesting that they do not activate head direction and conjunction cells within the hippocampal and para-hippocampal formations that are relevant for spatial learning (Thorndyke and Hayes-Roth, 1982; Moser et al., 2008). As a preliminary study, we developed a virtual reality task analogous to the MWM (VMWM) and the RAWM (VRAWM) tasks that are widely used in rodents. In the VMWM, we found that a test that included three trials with three extra-maze cues was optimal to assess spatial learning, while in the VRAWM, we found that a test that included two trials with two extra-maze cues was optimal to assess spatial learning.

Following 12 weeks of training, no significant differences were detected between control and exercise groups in the VMWM or VRAWM, when measuring success rate, latency, movement distance, speed, and path efficiency. Indeed, while a trend toward statistical significance was observed in parameters such as distance covered and movement speed in the VRAWM, the overall success rate or latency in this task was not significantly different between the two interventions groups.

Improvement in cardiovascular fitness correlates with the amelioration of cognitive functions (Heisz et al., 2017; Nauer et al., 2020). One of the markers for improving cardiovascular fitness is an improvement in VO_2max_ (Bacon et al., 2013; Lundby and Montero, 2019). Several studies showed that improving VO_2max_ parameters resulted in better performance in pattern separation tasks (Heisz et al., 2017; Nauer et al., 2020). We did not find any significant differences in spatial learning and memory between control and exercise groups. However, the VO_2max_ scores also did not change following the 12 weeks of a mild-intensity aerobic training program between the two groups. If cardiovascular fitness improvement is a key factor in the effect of exercise on cognitive abilities, it is no surprise that in our current study, we did not find improvement in spatial learning and memory, as the subjects who performed the training program did not increase their VO_2max_. Since the target position and the location of the extra-maze cues were identical for both the baseline and post-intervention phases, the component of long-term spatial memory of this task was larger in the post-intervention test compared with the baseline test. Thus, performance in the post-intervention task was affected by both learning and long-term memory. While we did not find a significant improvement in VO_2max_ after the training program, we did find that time to anaerobic threshold significantly improved following the training program between the two groups. The improvement in time to anaerobic threshold and the lack of improvement in VO_2max_ can suggest that VO_2max_ has a more important role in improving spatial learning and memory than time to anaerobic threshold.

The current study provides a new system for measuring spatial learning and memory in humans using VR. For studying the effect of aerobic exercise on spatial learning and memory, future studies may need to use a more intense training program to create improvement in VO_2max_ that may enhance spatial learning and memory.

## Data Availability Statement

The original contributions presented in the study are included in the article/Supplementary Materials, further inquiries can be directed to the corresponding author.

## Ethics Statement

The study was approved by the Bar-Ilan University's Ethics Committee in accordance with the Declaration of Helsinki, and by the institutional review boards (IRBs) of the Israel Defense Force's Medical Corps (1529-15) and the Sheba Medical Center (3321-16). Prior to testing, subjects were explained on the study's aims and required to sign informed consent forms, which were approved by all three IRBs. The patients/participants provided their written informed consent to participate in this study.

## Author Contributions

EO, RY, TB-Z, and IK: conceived and designed research. SA and IK: VO_2max_ test. TB-Z: VR test and analyze results of experiment. TB-Z and IW: designed virtual reality tests. RY, IK, and SA: designed the intervention program. TB-Z, EO, and RY: edited and revised manuscript. TB-Z and EO: approved final version of manuscript. All authors contributed to the article and approved the submitted version.

## Conflict of Interest

The authors declare that the research was conducted in the absence of any commercial or financial relationships that could be construed as a potential conflict of interest.

## Supplementary Material

Supplementary Figure 1 Comparison of baseline vs. post-intervention performance in the VMWM and VRAWM tests. We analyzed the differences in performance between baseline and post-intervention tests in latency to reach the hidden target in the VMWM and VRAWM: **(A)** VMWM latency to reach the target in the control group, **(B)** VMWM latency to reach the target in the exercise group, **(C)** VRAWM latency to reach the target in the control group, **(D)** VRAWM latency to reach the target in the exercise group. #: a significant main days effect; ^*^a significant main intervention effect.

Supplementary Figure 2 VO_2max_ and time to anaerobic threshold did not correlate with performance in both VMWM and VRAWM tasks. Correlation test was conducted between fitness performance and latency to reach the target in the last day post-intervention test in both VMWM and VRAWM. **(A)** VMWM latency to reach the target and VO2_max_, **(B)** VMWM latency to reach the target and time to anaerobic threshold, **(C)** VRAWM latency to reach the target and VO2_max_, **(D)** VRAWM latency to reach the target and time to anaerobic threshold (*p* > 0.05).

## References

[B1] Bacon A. P. Carter R. E. Ogle E. A. Joyner M. J. (2013). VO_2max_ trainability and high intensity interval training in humans: a meta-analysis. PLoS ONE 8:e73182. 10.1371/journal.pone.0073182 24066036 PMC3774727

[B2] Banta Lavenex P. A. Colombo F. Ribordy Lambert F. Lavenex P. (2014). The human hippocampus beyond the cognitive map: evidence from a densely amnesic patient. Front. Hum. Neurosci. 8:711. 10.3389/fnhum.2014.00711 25309387 PMC4164002

[B3] Barak B. Feldman N. Okun E. (2015). Cardiovascular fitness and cognitive spatial learning in rodents and in humans. J. Gerontol. A Biol. Sci. Med. Sci. 70, 1059–1066. 10.1093/gerona/glu162 25227128 PMC4536905

[B4] Burgess N. Maguire E. A. O'Keefe J. (2002). The human hippocampus and spatial and episodic memory. Neuron 35, 625–641. 10.1016/S0896-6273(02)00830-9 12194864

[B5] Caplan J. B. Madsen J. R. Schulze-Bonhage A. Aschenbrenner-Scheibe R. Newman E. L. Kahana M. J. (2003). Human theta oscillations related to sensorimotor integration and spatial learning. J. Neurosci. 23, 4726–4736. 10.1523/JNEUROSCI.23-11-04726.2003 12805312 PMC6740775

[B6] Cassilhas R. C. Tufik S. de Mello M. T. (2016). Physical exercise, neuroplasticity, spatial learning and memory. Cell. Mol. Life Sci. 73, 975–983. 10.1007/s00018-015-2102-0 26646070 PMC11108521

[B7] Eichenbaum H. (2015). The hippocampus as a cognitive map … of social space. Neuron 87, 9–11. 10.1016/j.neuron.2015.06.013 26139366

[B8] Eknoyan G. (2008). Adolphe quetelet (1796-1874)–the average man and indices of obesity. Nephrol. Dial. Transplant. 23, 47–51. 10.1093/ndt/gfm517 17890752

[B9] Fairshter R. D. Walters J. Salness K. Fox M. Minh V. D. Wilson A. F. (1983). A comparison of incremental exercise tests during cycle and treadmill ergometry. Med. Sci. Sports Exerc. 15, 549–554. 10.1249/00005768-198315060-00020 6656567

[B10] Hartley T. Lever C. Burgess N. O'Keefe J. (2014). Space in the brain: how the hippocampal formation supports spatial cognition. Philos. Trans. R. Soc. Lond. B. Biol. Sci. 369:20120510. 10.1098/rstb.2012.0510 24366125 PMC3866435

[B11] Heisz J. J. Clark I. B. Bonin K. Paolucci E. M. Michalski B. Becker S.. (2017). The effects of physical exercise and cognitive training on memory and neurotrophic factors. J. Cogn. Neurosci. 29, 1895–1907. 10.1162/jocn_a_01164 28699808

[B12] Herting M. M. Nagel B. J. (2012). Aerobic fitness relates to learning on a virtual morris water task and hippocampal volume in adolescents. Behav. Brain Res. 233, 517–525. 10.1016/j.bbr.2012.05.012 22610054 PMC3403721

[B13] Holzschneider K. Wolbers T. Röder B. Hötting K. (2012). Cardiovascular fitness modulates brain activation associated with spatial learning. Neuroimage 59, 3003–3014. 10.1016/j.neuroimage.2011.10.021 22027496

[B14] Howley E. T. Bassett D. R. Welch H. G. (1995). Criteria for maximal oxygen uptake: review and commentary. Med. Sci. Sports Exerc. 27, 1292–1301. 10.1249/00005768-199509000-00009 8531628

[B15] Iglói K. Doeller C. F. Berthoz A. Rondi-Reig L. Burgess N. (2010). Lateralized human hippocampal activity predicts navigation based on sequence or place memory. Proc. Natl. Acad. Sci. U. S. A. 107, 14466–14471. 10.1073/pnas.1004243107 20660746 PMC2922562

[B16] Lee M. C. Okamoto M. Liu Y. F. Inoue K. Matsui T. Nogami H.. (2012). Voluntary resistance running with short distance enhances spatial memory related to hippocampal BDNF signaling. J. Appl. Physiol. (1985) 113, 1260–1266. 10.1152/japplphysiol.00869.2012 22936723

[B17] Leveritt M. Abernethy P. J. Barry B. K. Logan P. A. (1999). Concurrent strength and endurance training. A review. Sports Med. 28, 413–427. 10.2165/00007256-199928060-00004 10623984

[B18] Levy L. J. Astur R. S. Frick K. M. (2005). Men and women differ in object memory but not performance of a virtual radial maze. Behav. Neurosci. 119, 853–862. 10.1037/0735-7044.119.4.853 16187814

[B19] Loprinzi P. D. (2018). Intensity-specific effects of acute exercise on human memory function: considerations for the timing of exercise and the type of memory. Health Promot Perspect. 8, 255–262. 10.15171/hpp.2018.36 30479978 PMC6249493

[B20] Lourenço T. F. Martins L. E. Tessutti L. S. Brenzikofer R. Macedo D. V. (2011). Reproducibility of an incremental treadmill VO(2)max test with gas exchange analysis for runners. J. Strength Cond. Res. 25, 1994–1999. 10.1519/JSC.0b013e3181e501d6 21487313

[B21] Lundby C. Montero D. (2019). Did you know-why does maximal oxygen uptake increase in humans following endurance exercise training? Acta Physiol. 227:e13371. 10.1111/apha.13371 31465612

[B22] Moser E. I. Kropff E. Moser M. B. (2008). Place cells, grid cells, and the brain's spatial representation system. Annu. Rev. Neurosci. 31, 69–89. 10.1146/annurev.neuro.31.061307.090723 18284371

[B23] Nagamatsu L. S. Chan A. Davis J. C. Beattie B. L. Graf P. Voss M. W.. (2013). Physical activity improves verbal and spatial memory in older adults with probable mild cognitive impairment: a 6-month randomized controlled trial. J. Aging Res. 2013:861893. 10.1155/2013/861893 23509628 PMC3595715

[B24] Nauer R. K. Dunne M. F. Stern C. E. Storer T. W. Schon K. (2020). Improving fitness increases dentate gyrus/CA3 volume in the hippocampal head and enhances memory in young adults. Hippocampus 30, 488–504. 10.1002/hipo.23166 31588607 PMC7485880

[B25] Piber D. Nowacki J. Mueller S. C. Wingenfeld K. Otte C. (2018). Sex effects on spatial learning but not on spatial memory retrieval in healthy young adults. Behav. Brain Res. 336, 44–50. 10.1016/j.bbr.2017.08.034 28847444

[B26] Ploughman M. (2008). Exercise is brain food: the effects of physical activity on cognitive function. Dev. Neurorehabil. 11, 236–240. 10.1080/17518420801997007 18781504

[B27] Possin K. L. Sanchez P. E. Anderson-Bergman C. Fernandez R. Kerchner G. A. Johnson E. T.. (2016). Cross-species translation of the Morris maze for Alzheimer's disease. J. Clin. Invest. 126, 779–783. 10.1172/JCI78464 26784542 PMC4731157

[B28] Prentice A. M. Jebb S. A. (2001). Beyond body mass index. Obes. Rev. 2, 141–147. 10.1046/j.1467-789x.2001.00031.x 12120099

[B29] Schoenfeld R. Schiffelholz T. Beyer C. Leplow B. Foreman N. (2017). Variants of the Morris water maze task to comparatively assess human and rodent place navigation. Neurobiol. Learn. Mem. 139, 117–127. 10.1016/j.nlm.2016.12.022 28057502

[B30] Snider J. Plank M. Lee D. Poizner H. (2013). Simultaneous neural and movement recording in large-scale immersive virtual environments. IEEE Trans. Biomed. Circuits Syst. 7, 713–721. 10.1109/TBCAS.2012.2236089 24232632

[B31] Tan H. M. Wills T. J. Cacucci F. (2017). The development of spatial and memory circuits in the rat. Wiley Interdiscip. Rev. Cogn. Sci. 8:e1424. 10.1002/wcs.1424 27943643

[B32] Thorndyke P. W. Hayes-Roth B. (1982). Differences in spatial knowledge acquired from maps and navigation. Cogn. Psychol. 14, 560–589. 10.1016/0010-0285(82)90019-6 7140211

[B33] van Praag H. (2009). Exercise and the brain: something to chew on. Trends Neurosci. 32, 283–290. 10.1016/j.tins.2008.12.007 19349082 PMC2680508

[B34] Voss M. W. Vivar C. Kramer A. F. van Praag H. (2013). Bridging animal and human models of exercise-induced brain plasticity. Trends Cogn Sci. 17, 525–544. 10.1016/j.tics.2013.08.001 24029446 PMC4565723

[B35] Weltman A. Snead D. Stein P. Seip R. Schurrer R. Rutt R.. (1990). Reliability and validity of a continuous incremental treadmill protocol for the determination of lactate threshold, fixed blood lactate concentrations, and VO_2max_. Int. J. Sports Med. 11, 26–32. 10.1055/s-2007-1024757 2318561

[B36] Witter M. P. Wouterlood F. G. Naber P. A. Van Haeften T. (2000). Anatomical organization of the parahippocampal-hippocampal network. Ann. N. Y. Acad. Sci. 911, 1–24. 10.1111/j.1749-6632.2000.tb06716.x 10911864

[B37] Woost L. Bazin P. L. Taubert M. Trampel R. Tardif C. L. Garthe A.. (2018). Physical exercise and spatial training: a longitudinal study of effects on cognition, growth factors, and hippocampal plasticity. Sci. Rep. 8:4239. 10.1038/s41598-018-19993-9 29523857 PMC5844866

[B38] Xu D. Hao X. Wang Z. Duan Y. Liu F. Marsh R. (2012). A virtual radial arm maze for the study of multiple memory systems in a functional magnetic resonance imaging environment. Int. J. Virtual Real. 11, 63–76. 10.20870/IJVR.2012.11.2.2844 26366052 PMC4564131

