# Supplementary figures and images for: Mild Physical Activity Does Not Improve Spatial Learning in a Virtual Environment
Source: Front Behav Neurosci. 2020 Nov 17;14:584052. doi: 10.3389/fnbeh.2020.584052 (PMC7705229; doi:10.3389/fnbeh.2020.584052)

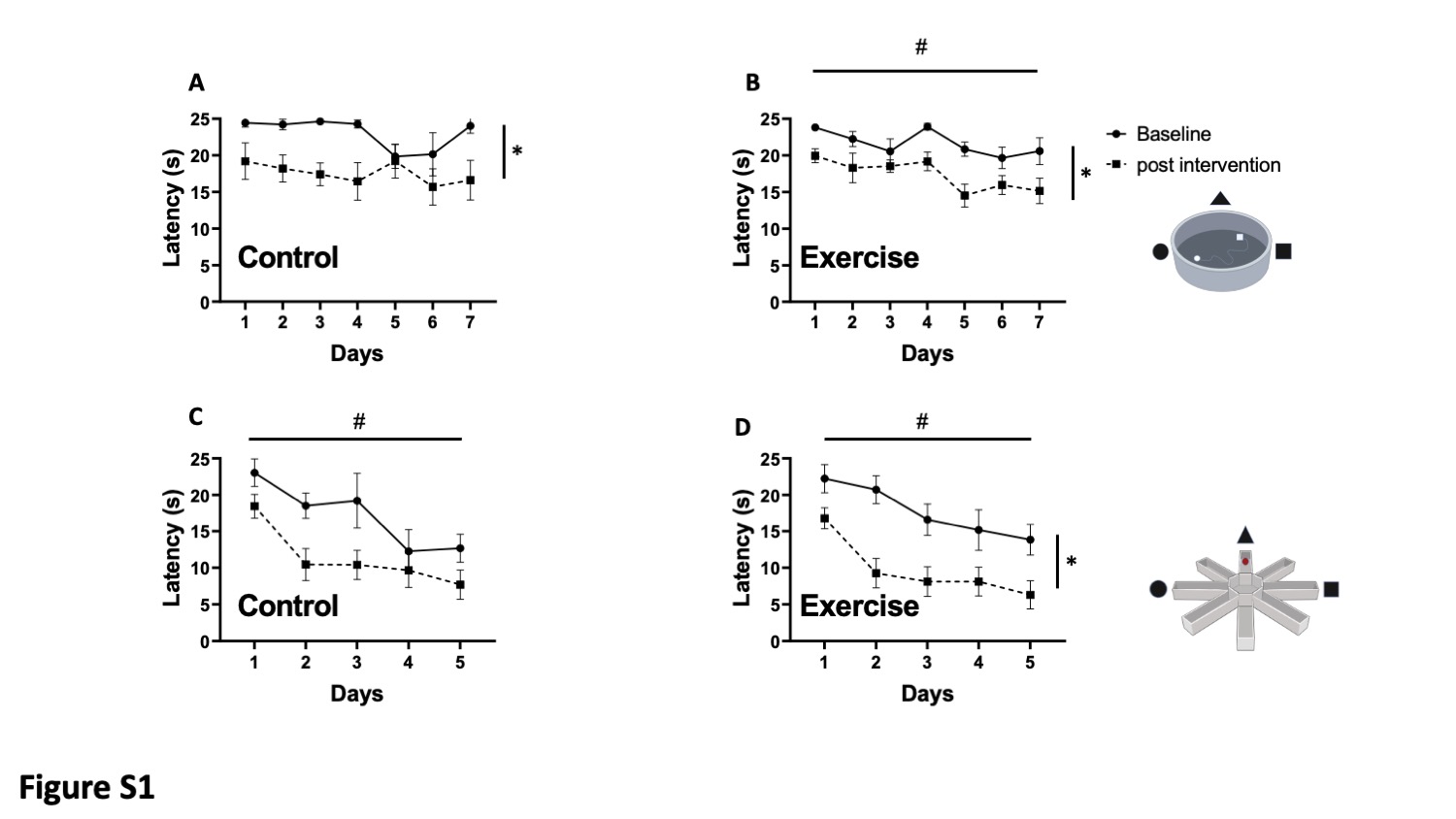

Supplement: Supplementary Figure 1 — Comparison of baseline vs. post-intervention performance in the VMWM and VRAWM tests. We analyzed the differences in performance between baseline and post-intervention tests in latency to reach the hidden target in the VMWM and VRAWM: (A) VMWM latency to reach the target in the control group, (B) VMWM latency to reach the target in the exercise group, (C) VRAWM latency to reach the target in the control group, (D) VRAWM latency to reach the target in the exercise group. #: a significant main days effect; *a significant main intervention effect. [file Image_1.JPEG]

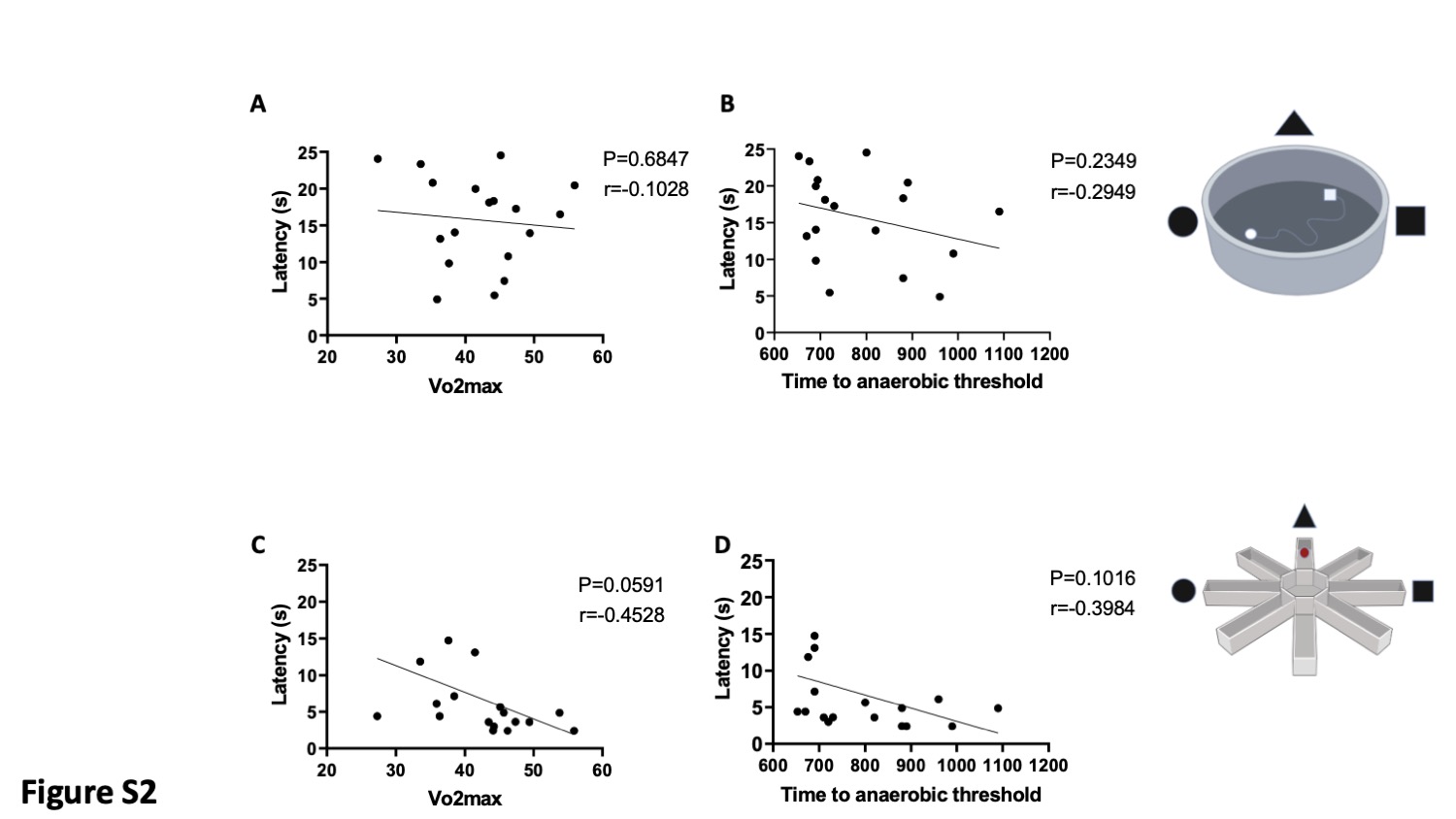

Supplement: Supplementary Figure 2 — VO2max and time to anaerobic threshold did not correlate with performance in both VMWM and VRAWM tasks. Correlation test was conducted between fitness performance and latency to reach the target in the last day post-intervention test in both VMWM and VRAWM. (A) VMWM latency to reach the target and VO2max, (B) VMWM latency to reach the target and time to anaerobic threshold, (C) VRAWM latency to reach the target and VO2max, (D) VRAWM latency to reach the target and time to anaerobic threshold (p > 0.05). [file Image_2.JPEG]
